# Localised childhood vulvar pemphigoid: a rare case study

**DOI:** 10.52054/FVVO.13.3.031

**Published:** 2021-09-24

**Authors:** AS Peeters, N Dhont, H Stals

**Affiliations:** Department of Obstetrics and Gynaecology, ZOL Hospitals, Schiepse Bos 6, 3600 Genk, Belgium

**Keywords:** bullous pemphigoid, childhood dermatitis, topical corticosteroids, vulvar pemphigoid

## Abstract

In this case report we present a young patient with localised childhood vulvar pemphigoid. It is a rare variant of bullous pemphigoid with mostly a favourable prognosis and prompt response to potent topical corticosteroids. She presented with relapsing vulvar pain and lesions. Our case enlightens the recognition of this unusual subtype and the importance of performing a cutaneous biopsy.

## Introduction

Bullous pemphigoid (BP) is an autoimmune subepidermal bullous dermatitis, it affects primarily the elderly with a median age of onset from 60 to 75 years (Korman, 1987; Fisler et al., 2003). However BP can also rarely occur in childhood, they have a favourable evolution and the lesions are often more localised (Laffitte and Borradori , 2001; Fisler et al., 2003).

Vulvar involvement is more frequently observed with children then adults (40% vs 9%). Localised childhood vulvar pemphigoid (LCVP) is a morphologic variant of bullous pemphigoid and occurs with non-scarring, recurrent vesicles and erosions confined to the vulva which show a good response to topical steroids (Saad et al., 1992; Farrell et al., 1999). This entity should be recognised in the differential diagnosis of persistent localised vulvar erosions in this specific population.

We here describe the case of a 12 year old girl presenting with vulvar lesions. Several diagnostic tests were performed and the diagnosis of LCVP was confirmed by biopsy. She had a favourable response to topical steroids.

## Case presentation

A young girl of 12 years old presented with vulvar irritation and itching at our general gynaecology outpatient clinic. The patient was in good health and took no medication. She had presented with these symptoms to her general practitioner a few weeks before. A vaginal swab was taken and a miconazole ointment was prescribed. As symptoms did not improve she presented a few days later to the emergency department of a neighbouring hospital and was given a miconazole and zinc oxide ointment.

Two weeks later when she presented in our centre we observed erythema and irritation around the introitus, without blisters. The remaining cutaneous examination was entirely normal. Continuation of miconazole and zinc oxide ointment with the addition of Vaseline was suggested since symptoms had somewhat improved with this.

**Figure 1:**
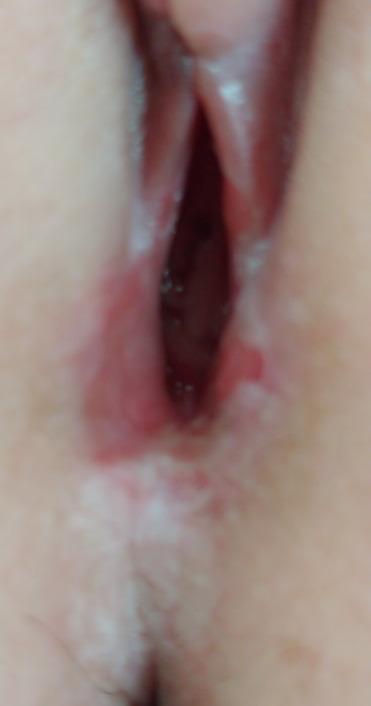
Localised vulvar erosion upon examination

After 8 days she came back to the hospital because of an enlargement of the vulvar lesion and dysuria with urinary retention. Clinical examination showed a blistering ulcer of 5mm at the introitus, easily bleeding and painful on palpation (Figure 1). Differential diagnosis included genital herpes and a Lipschütz ulcer. A swab for PCR was taken, the result was negative for Herpes simplex DNA. Treatment with a corticosteroid ointment and a local anaesthetic gel to facilitate micturition was started.

Upon follow up 5 days later the vulvar erosion had resolved and treatment was continued for 1 week. Two weeks later a new erosive vestibular lesion had developed. A swab for Herpes simplex and Varicella zoster PCR was performed again, no detection of either DNA was found. A full serology blood sample was taken, this showed a previous Epstein-Barr infection, Herpes simplex type 1 and type 2 IgG negative and no immunisation for cytomegalovirus. The corticosteroid ointment treatment was prolonged.

**Figure 2:**
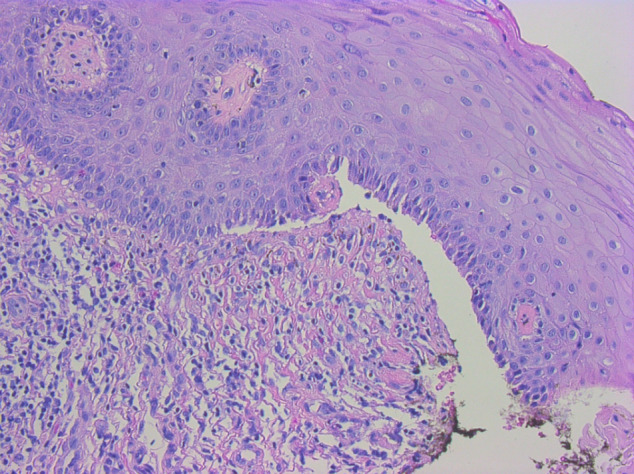
Histology section showing a sub epidermal bullae (thin arrow) and infiltration with eosinophils (thick arrow).

Finally, she was referred to our multi-disciplinary vulva outpatient clinic for further investigation. In this clinic patients are examined by both a dermatologist and a gynaecologist. A biopsy under sedation was planned for a differential diagnosis of herpes, bullous disease or contact dermatitis. We took 2 punch biopsies with one specimen sent fresh to the laboratory for immunofluorescence testing, the result was negative. Histologic examination of the second specimen showed a subepidermal blister forming and mixed infiltration with neutrophils and eosinophils which was suggestive for a localised vulvar pemphigoid (Figure 2). A treatment with local application of topical corticosteroids, betamethasone dipropionate 16 mg in hydrophilic cream, was started.

We did a check-up after 6 weeks and this showed a good response to the treatment, no more lesions were visualised. We suggested a discontinuation of the topical corticosteroids.

## Discussion

BP is more frequent in adults and rarely affects children. Therefore, it is important to know the difference in behaviour between both entities. In children there is a milder manifestation with more localised lesions, a quick response to topical corticosteroids and rare relapses (Fisler et al., 2003).

There are two peak ages for childhood BP (Waisbourd-Zinman et al., 2008). The infantile form occurs during the first year of life, the childhood form arises around 8 years (Santos et al., 2007). The childhood form shows genital involvement in 44% of the cases, isolated or not (Waisbourd- Zinman et al., 2008). Localised childhood vulvar pemphigoid (LCVP) is an excessively rare variant of the childhood form of the disease (Saad et al., 1992), with only 12 cases reported in the literature. BP is an autoimmune disease. It is characterised by tissue-bound and circulating autoantibodies against structural proteins (BP180 and BP 230 antigens) of the hemidesmosomes, structures connecting basal keratinocytes with the basement membrane, and subepidermal blistering (Laffitte and Borradori, 2001; Di Zenzo et al., 2008). A diagnosis can be made based on histology and immunofluorescence (IF), the latter requires a fresh biopsy specimen. The direct IF shows linear IgG (50-90% of cases) and C3 deposits at the cutaneous basement membrane zone (80-100% of cases) (Guenther and Shum, 1990; Saad et al., 1992). Indirect IF demonstrates circulating IgG directed against some components of the basement membrane (in 70% of the cases) (Guenther and Shum, 1990). In our case these IgG were not found. The characteristic histology of the lesions includes a subepidermal vesicle associated with a dermal inflammatory infiltrate rich in eosinophils (Holubar, 1984). The detection of this typical histological image confirmed our diagnosis of LCVP (Figure 2).

The differential diagnosis of vulvar blistering and erosions during childhood include various entities. Examining the actual literature, we listed the entities to keep in mind while facing a child with unremitting vulvar erosions (DeCastro et al., 1985; Marren et al., 1993; Bouloc et al., 1998; Farrell et al., 1999; Schumann et al., 1999; Fischer and Rodgers, 2000).

- Bullous lichen sclerosus et atrophicus
- Erythema multiforme/Stevens-Johnson syndrome
- Epidermolysis bullosa acquisita (EBA)
- Bullous lupus erythematosus
- Bullous impetigo
- Herpes simplex
- Bullous fixed drug eruption
- Child abuse
- Lichen planus pemphigoid (LPP)
- Localized pemphigoid of childhood
- Streptococcal vulvovaginitis
- Lipschütz ulcer

The clinical course of LCVP is usually favourable with a good response to topical steroids. Other treatments such as antimicrobials, dapsone, systemic steroids or immunosuppressive agents have been used to control the disease and prevent development of scarring (Marren et al., 1993; Farrell et al., 1999). If our patient had not responded well to topical steroids we would have started a trial of systematic dapsone with blood monitoring because of the haemolysis risk (Gürcan and Ahmed, 2009).

Vulvar lesions in childhood are a challenging condition for clinicians and parents. Genital dermatoses are rare in children and are at the crossroads of 3 medical specialities: dermatology, gynaecology and paediatrics. In our case diagnosis was only reached when a multidisciplinary team consisting of a dermatologist, gynaecologist and pathologist worked together.

## Conclusion

Localised childhood vulvar pemphigoid is a rare presentation of bullous pemphigoid. The localised form has the best prognosis and responds well to topical steroids. When a young girl presents with localised vulvar erosions this entity needs to be recognised and diagnosis should be confirmed with histology and immunofluorescence performed on a fresh cutaneous biopsy.
